# Broadband and Broad-angle Polarization-independent Metasurface for Radar Cross Section Reduction

**DOI:** 10.1038/srep40782

**Published:** 2017-01-20

**Authors:** Hengyi Sun, Changqing Gu, Xinlei Chen, Zhuo Li, Liangliang Liu, Bingzheng Xu, Zicheng Zhou

**Affiliations:** 1 Key Laboratory of Radar Imaging and Microwave Photonics, Ministry of Education, College of Electronic and Information Engineering, Nanjing University of Aeronautics and Astronautics, Nanjing 210016, China; 2 State Key Laboratory of Millimeter Waves, Southeast University, Nanjing 210096, China

## Abstract

In this work, a broadband and broad-angle polarization-independent random coding metasurface structure is proposed for radar cross section (RCS) reduction. An efficient genetic algorithm is utilized to obtain the optimal layout of the unit cells of the metasurface to get a uniform backscattering under normal incidence. Excellent agreement between the simulation and experimental results show that the proposed metasurface structure can significantly reduce the radar cross section more than 10 dB from 17 GHz to 42 GHz when the angle of incident waves varies from 10° to 50°. The proposed coding metasurface provides an efficient scheme to reduce the scattering of the electromagnetic waves.

Engineered metamaterials and metasurfaces usually comprise periodic or non-periodic subwavelength unit cells that enable the manipulation of electromagnetic wave propagation to obtain unusual properties^1^,^2^,^3^ such as negative refraction^4^,^5^,^6^, subwavelength focusing^7^,^8^,^9^, and electromagnetic invisibility cloaking^10^,^11^,^12^,^13^,^14^,^15^,^16^. Among these examples, electromagnetic invisibility cloaking represents a prominent utilization of metamaterials for greatly reducing the radar cross section (RCS) of targets in military applications^17^,^18^. A perfect invisibility cloak presents no backward or forward RCS, and the optical transformation method provides an efficient means of bending incident electromagnetic waves along a given region for this purpose^19^,^20^,^21^. A chessboard-like structure combining perfect electric conductors and artificial magnetic conductors (AMCs) has been employed to reduce the RCS effectively^22^. However, it is difficult to increase the bandwidth of AMC structures due to AMC resonance. Therefore, broadening the working bandwidth of a metasurface for RCS reduction is a major issue that should be addressed. Another method of reducing the RCS is scattering cancellation. In this case, the metamaterial is homogeneous, and leads to the polarization of the incident wave converted to its cross-polarized one, which represents the plasmon cloaking of an object^23^,^24^,^25^. Recently, a new approach to reducing the scattering of electromagnetic waves using coding metasurfaces composed of digital coding elements has been proposed, and a 1-bit, 2-bit, and 3-bit random coding metamaterial has been reported^26^. Far-field scattering pattern analysis in conjunction with a hybrid optimization algorithm has been employed to obtain an optimal arrangement of digital coding elements for designing a low RCS metasurface with ultra-low backward scattering that is functional over a broadband from 7 GHz to 14 GHz^27^. In addition, the particle-swarm optimization algorithm has been employed to establish the optimal coding sequences of Minkowski closed loop particles for reducing the scattering of terahertz waves^28^.

Genetic algorithms represent another well established optimization method employed in electromagnetic applications. A GA is an optimization technique that searches for optimal solutions by simulating natural selection processes and the genetic mechanism of the biological evolutionary process. The technique was first proposed by Holland in 1975^29^. As early as the 1990s, Haupt^30^,^31^ significantly employed GAs in electromagnetic research. Twenty years later, Johnson^32^ employed a GA to optimize the design of one-dimensional (1D) and two-dimensional (2D) array antennas, and the optimization process was shown to overcome the limitations associated with conventional optimization techniques when applied to antenna arrays. Marcano^33^,^34^,^35^,^36^,^37^ applied GAs in the design of linear and plane array antennas. Application of GAs for implementing RCS reduction has also been pursued. For example, Mosallaei^38^ combined a GA with the mode equation, obtained in a wide frequency band, and applied the method to effectively minimize the RCS of a radar absorbing material (RAM). Zhu^39^ combined a standard GA with the high frequency method to optimize RCS reduction by changing the normal anisotropy surface impedance distribution of a geometric structure for missile applications. Yao^40^ obtained the dimensions of a metallic patch by means of GA optimization, and successfully designed a low RCS patch antenna. Thus, the application of GAs to electromagnetic research is quite mature, and the use of a GA to design a low RCS antenna array is certainly feasible.

In the present study, a random 1-bit digital coding metasurface for RCS reduction is designed based on diffuse reflection theory, and a wideband property is imparted to the metasurface by means of wideband cross polarization conversion^41^. The proposed metasurface exhibits broadband, broad-angle, and polarization-independent for RCS reduction. Rather than employing multi-bit elements, the necessary phase difference can be obtained with 1-bit elements by simply rotating the structures, and the proposed metasurface therefore consists of unit cells with “0” and “1” elements owing to their ***0*** and ***π*** phase responses. Furthermore, an efficient strategy for designing random coding metasurfaces is developed by employing a combination of diffuse reflection theory and the scattering pattern reshaping method in conjunction with a genetic algorithm (GA) to optimize the sequences of the unit cells of the non-periodic random metasurface structure. Both simulation and experimental measurement results indicate that the optimized random coding metasurface reduces the RCS by greater than 10 dB over a frequency range of 17–42 GHz for both ***x-*** and ***y-polarized*** normal and obliquely incident electromagnetic waves, and the bandwidth is not reduced at all under oblique incidence. Both experimental and simulation results verify the reliability and validity of the proposed method.

## Results

### Unit cell properties and optimal sequencing of the metasurface

**Figure 1:**
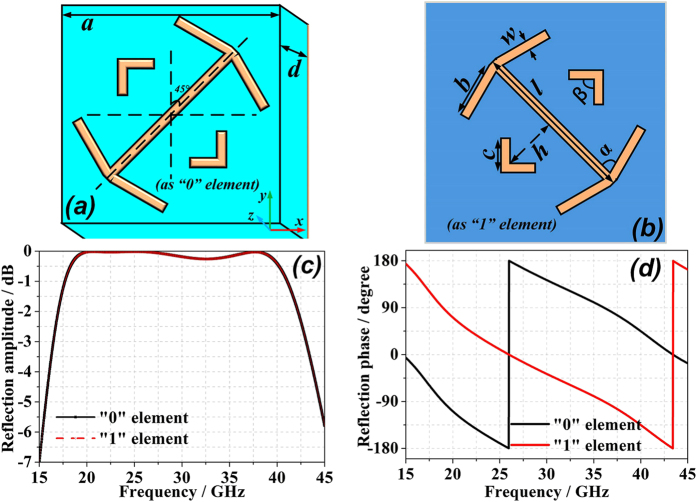
Front views of “0” and “1” element unit cells. The unit cell structure with an angle of 45° to the y axis represents the “0” element **(a)**, and the structure rotated 90° counter-clockwise about the z axis is the “1” element **(b)**. Reflection properties of “0” and “1” elements versus frequency under normal x-polarized electromagnetic incident waves: **(c)** lattice reflection amplitudes; **(d)** lattice reflection phases.

**Figure 2:**
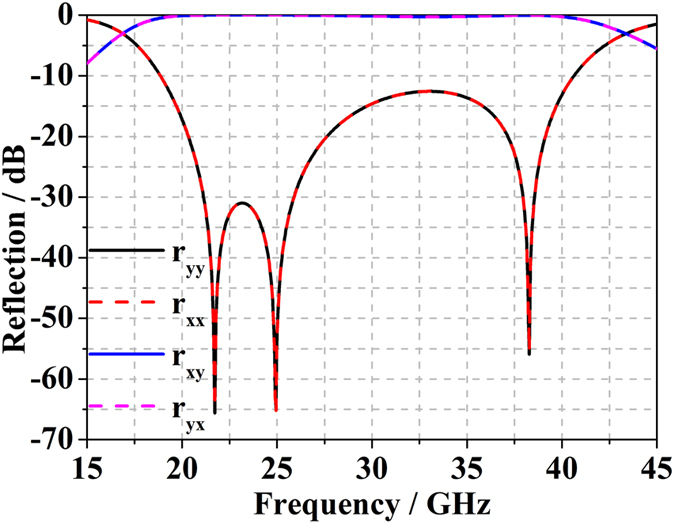
Simulated reflections of the unit cell under normal x- and y-polarized electromagnetic incident waves.

In general, the unit cell structure of a polarization conversion metasurface exhibits different phase responses under normal incidence (along the ***z-direction***) depending on the rotation angle in the***xy-plane***. Two structures with relative phase responses of ***π*** can be arranged in a chessboard-like metasurface to reduce the RCS under monostatic backscattering conditions. In the worst case, the reflection consists of four strong lobes, which can be easily detected by bistatic detection. A 1-bit digital coding metasurface was proposed by Cui *et al*. in 2014^26^, which included two types of unit cells with ***0*** and ***π*** phase responses to mimic the “0” and “1” elements, respectively, and RCS reduction was achieved through the arbitrary arrangement of the unit cells. Inspired by this concept, we designed a new structure by simply rotating the original structure by ***90***°, which also exhibits broadband, broad-angle, and polarization-independent characteristics. The original structure and the new structure are illustrated in Fig. 1(a,b), respectively, in which the F4B-2 woven glass polytetrafluoroethylene dielectric substrate has a thickness ***d** = 1.5* mm with a dielectric constant ε_***r***_ *=* *2.65* and ***tanδ*** = *0.001*. The geometrical dimensions of the structure are ***a*** = 3 mm, ***w*** = 0.2 mm, ***l*** = 2.6 mm, ***b*** = 1.5 mm, ***c*** = 0.45 mm, ***h*** = 0.6 mm, ***α*** = 75°, ***β*** = 90° and the thickness of the metallic coating (PEC) ***t*** = 0.018 mm. The polarization conversion mechanism owing to the multiple resonances of the unit cells, and the reflection phase difference between the “0” and “1” unit cells remains ***π*** over the entire working band. Figure 2 illustrates the simulated reflection of the unit cell for normal ***x-*** and ***y-polarized*** incidence, in which ***R***_***xx***_***/R***_***yy***_ and ***R***_***yx***_***/R***_***xy***_ represent the reflection coefficients of the co-polarized and cross-polarized waves, respectively. Therefore, according to the work of Cui *et al*.^28^, we can easily design the coding metasurface using only two elements. To satisfy the periodic boundary required for element simulation, a lattice of dimension ***D***, containing 6 × 6 unit cells of equivalent orientation, is generated as the “0” or “1” element for the metasurface.

The simplest method of constructing an RCS-reducing metasurface using 1-bit unit cells is to generate a phase-distribution matrix with “0” and “1” elements randomly distributed, and place each element according to its reflection phase. However, a randomly constructed matrix does not necessarily exhibit the optimal phase distribution, and the resulting metasurface is not necessarily the best possible configuration. Hence, we adopt a GA to obtain the optimal configuration from a random sequence, and to ensure that the final design has the desired RCS reduction characteristics. We consider an *M* × *N* dimensional random permutation matrix containing digital 0 and 1 components, which represent the “0” and “1” lattices with the respective reflection phases ***0*** and ***π***. According to array theory, the lattice-scattered electric-field intensity in the far-field region can be expressed as


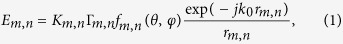


where, for *m* = 1, …, *M* and *n* = 1, …, *N, K*_*m,n*_ is the scale coefficient, *r*_*m,n*_ is the distance between the lattice point (*m, n*) and the far-field region observation point, and *f*_*m,n*_ (*θ, φ*) is the lattice scattering pattern function, given with respect to the elevation and azimuth angles ***θ*** and ***φ***. Moreover, 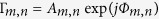, where *A*_*m,n*_ and *Φ*_*m,n*_ are the lattice reflection amplitude and phase coefficient, respectively, which are shown in Fig. 1(c,d) for “0” and “1” elements. We then define an array pattern function *F (θ, φ*) for the *M* × *N* lattice based on Eq. (1). It is usually safe to omit the effect of *K*_*m,n*_ and *f*_*m,n*_ (*θ, φ*) for a reflective coding metasurface when calculating *F(θ, φ*). In this analysis, we only consider the phase difference between the “0” and “1” elements, and assume that *A*_*m,n*_ is fixed to simplify the calculation. Thus, *F (θ, φ*) for the metasurface can be described as


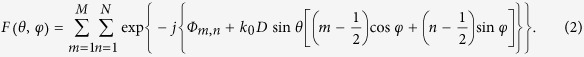


Then, we employ a GA to configure an optimal element arrangement for the random coding metasurface.

**Figure 3:**
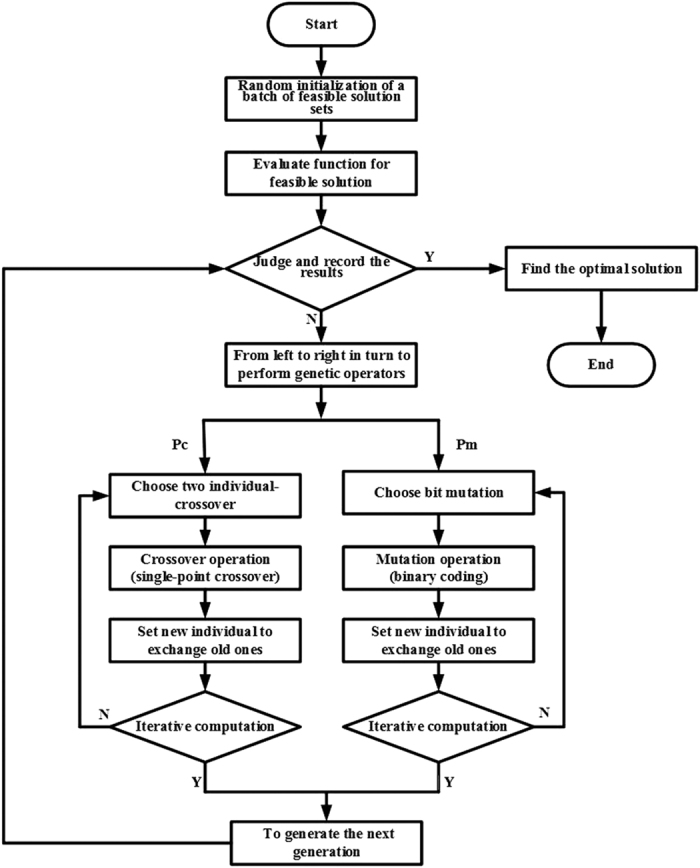
Flowchart of the genetic algorithm employed to search for the optimal unit cell configuration.

**Figure 4:**
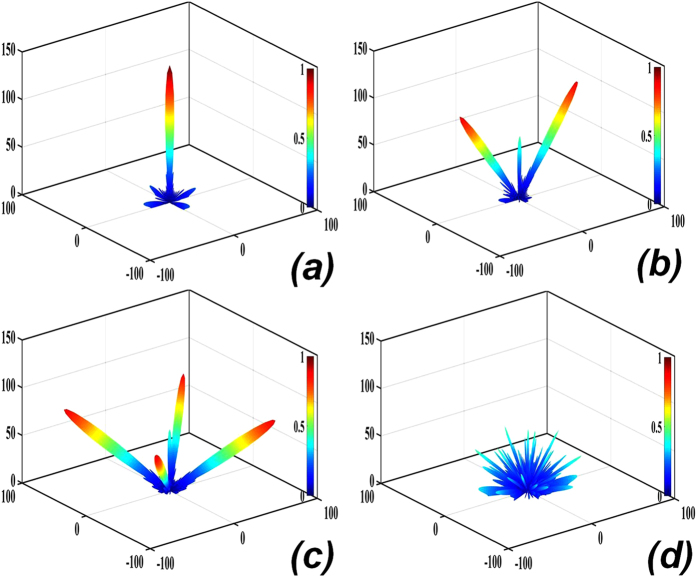
The normalized field intensity patterns obtained at 25.6 GHz for coding metasurfaces with coding sequences **(a)** 000000…/000000… (or 111111…/111111…, which yields equivalent results), **(b)** 010101…/010101…, **(c)** 010101…/101010…, and **(d)** the coding sequence obtained by the proposed optimization procedure. We see that the main lobe in the case of **(d)** is obviously decreased, and has produced many side lobes.

A flowchart of the GA applied in the present study is given in Fig. 3. The random initialization of a batch of feasible solution sets (*N*_*p*_) comprising 100 *M × N*, matrices, where *M = N = 12*, which consist of an arbitrary number of “0” and “1” elements distributed at random positions, represents the first generation of the search population. Equation (2) is employed as the evaluation function, and a threshold value of −10 dB is set to obtain an optimally high RCS reduction. Then, we establish uniform sampling points based on the values of ***θ*** and ***φ***according to 1° intervals to simplify the calculation, and generate 100 matrices with dimensions *D*_*θ*_ × *D*_*φ*_, where *D*_*θ*_ = 91 and *D*_*φ*_ = 361. Subsequently, we compute the sequence of matrices according to the value obtained from Eq. (2) from small to large. If the result meets the requirements, it is recorded. To facilitate the calculation, we employ fixed values of the crossover and mutation probabilities *p*_*c*_ = 0.9 and *p*_*m*_ = 0.1, respectively. In our GA, we employ single-point crossover as the most common crossover operator and binary mutation as the mutation operator. Then, the optimal solution is discovered by conducting 1000 iterative computations of the GA. After each iteration, the results are updated. After completion, we obtain the arrangement of “0” and “1” elements providing the minimum value of Eq. (2), which represents the metasurface providing the greatest RCS reduction. The normalized field intensity patterns obtained for coding metasurfaces with various uniform coding sequences lead to either a strong reflection beam or lobes projected toward the normal direction, as shown in Fig. 4(a–c). Comparing these figures with Fig. 4(d) obtained for the metasurface optimized according to the proposed procedure indicates that the optimized solution provides an ideal scattering reflection pattern.

### Simulations and measurement results

**Figure 5:**
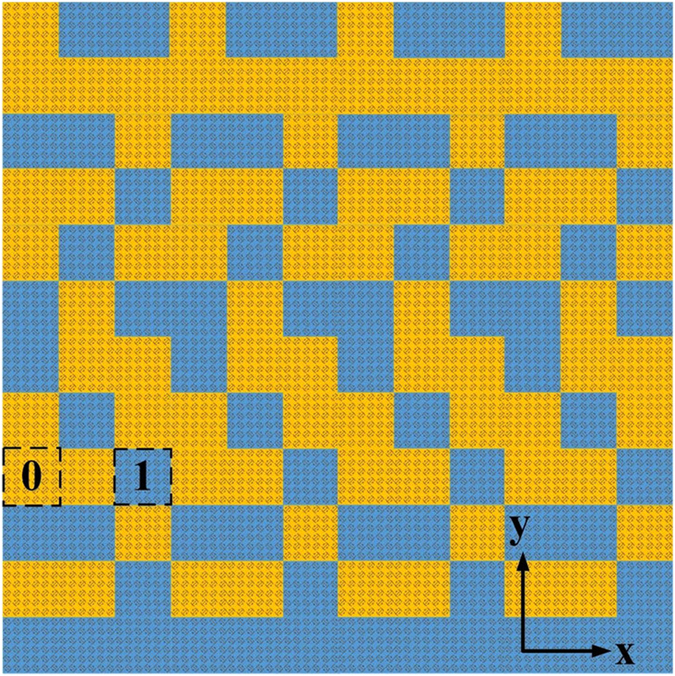
The optimized 1-bit coding metasurface of planform obtained by the GA. The “0” and “1” lattices comprise 6 × 6 equivalent unit cells, and are distinguished by yellow and blue colors, respectively.

**Figure 6:**
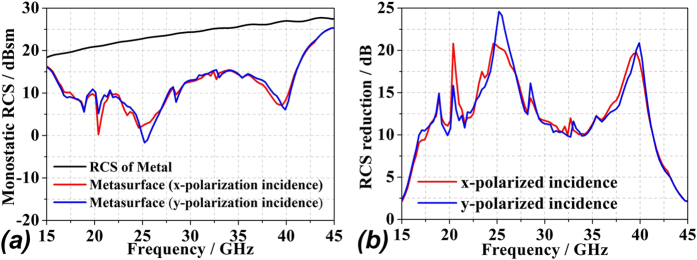
Simulated results of the metal surface and optimized coding metasurface under x- and y-polarized normal electromagnetic incident waves: **(a)** simulated monostatic RCS; **(b)** the RCS reduction.

**Figure 7:**
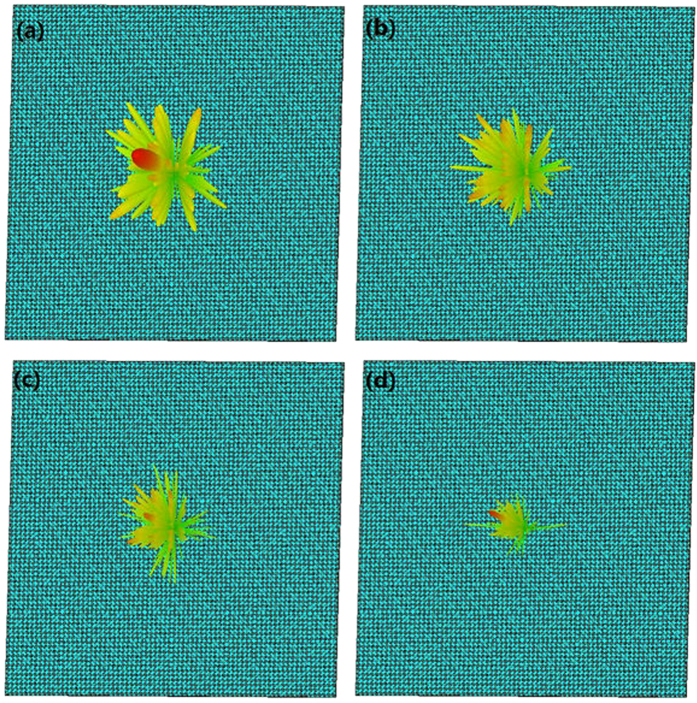
Three-dimensional scattering patterns of the optimized coding metasurface for normal x-polarized electromagnetic incident waves at **(a)** 15 GHz, **(b)** 25.6 GHz, **(c)** 34.2 GHz, and **(d)** 45 GHz.

**Figure 8:**
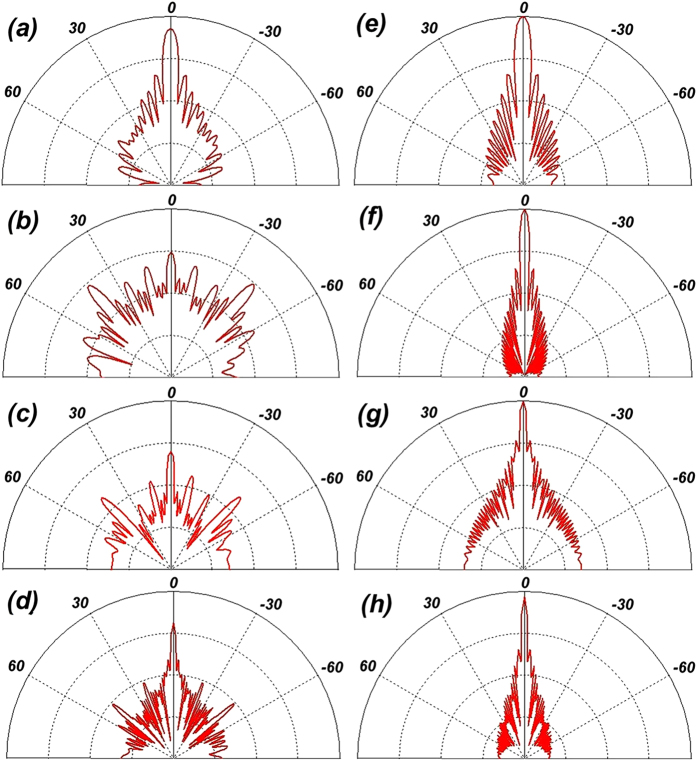
Scattering patterns of the optimized coding metasurface in the XOZ-plane at **(a)** 15 GHz, **(b)** 25.6 GHz, **(c)** 34.2 GHz, and **(d)** 45 GHz. Scattering patterns of the metal surface in the XOZ-plane at **(e)** 15 GHz, **(f)** 25.6 GHz, **(g)** 34.2 GHz, and **(h)** 45 GHz.

**Figure 9:**
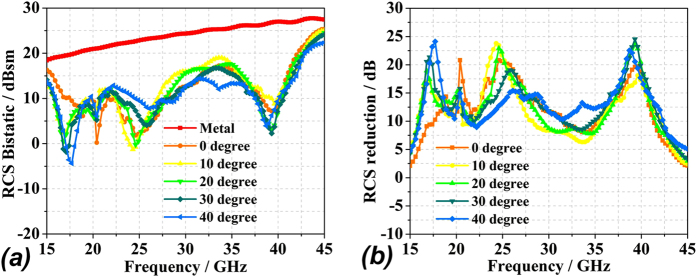
RCS of the metal and optimized coding metasurface under obliquely incident electromagnetic waves from 10° to 40°: **(a)** bistatic RCS; **(b)** RCS reduction.

Simulations of the optimal coding metasurface obtained by GA shown in Fig. 5 were conducted using CST Microwave Studio. For comparison, identical simulations were conducted with a metal (PEC) surface of equivalent geometry to that of the metasurface. When both ***x*****-** and ***y-polarized*** waves are normally incident on the metal surface and metasurface, the RCS appears as given in Fig. 6(a). Figure 6(b) shows a broadband 10 dB reduction in the RCS of the metasurface over frequency ranges from 17 GHz to 42 GHz. The RCS reduction between 24 GHz and 26 GHz is slightly more than 20 dB. A broadband RCS reduction more than 15 dB is observed for a 4 GHz bandwidth from 23.1 GHz to 27.1 GHz, and the RCS reduction presents a maximum of 25 dB at 25.6 GHz under y-polarized electromagnetic incident waves. Figure 7(a–d) show the simulated three-dimensional (3D) scattering patterns of the metasurface at 15 GHz, 25.6 GHz, 34.2 GHz, and 45 GHz, respectively. The radiation patterns shown in the Fig. 4(d) are in agreement with the full-wave simulations at 25.6 GHz. To compare the scattering properties of the metasurface and the metal surface quantitatively, the scattering patterns in the ***xz-plane*** obtained along a line passing through the middle of the surfaces (i.e., y = 0 [the ***XOZ-plane***]) were obtained by simulation at 15 GHz, 25.6 GHz, 34.2 GHz, and 45 GHz, and the patterns for the metasurface and the metal surface are shown in Fig. 8(a–h), respectively. According to the law of energy conservation, the RCS reduced metasurface must have a repressed main lobe and enforced side lobes to maintain the overall scattering energy. Indeed, it can be observed in Fig. 8(e–h) that a strong main lobe appears in the backward direction for the metal surface in the full working band. In Fig. 8(b,c,f,g), the metasurface is observed to have suppressed the main lobe energy, and uniformly scattered energy to the side lobes at 25.6 GHz and 34.2 GHz relative to the metal surface. To investigate the scattering properties of the metasurface under oblique incidences, electromagnetic waves impinging from 10°, 20°, 30°, and 40° were considered. Due to the properties of the unit cells, linear polarization conversion can be realized over a wide range of oblique incidence angles. The RCS and RCS reduction over a broadband at the incidence angles considered can be observed in Fig. 9(a,b), respectively.

**Figure 10:**
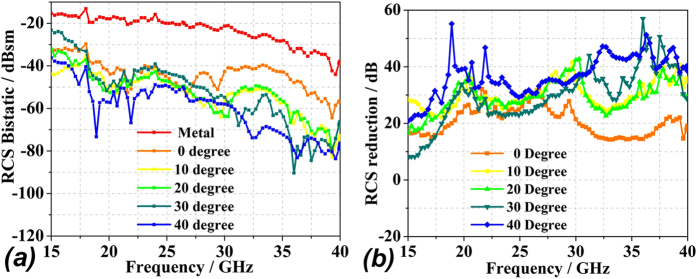
The experimentally obtained RCS **(a)** and RCS reduction **(b)** for various incident angles with vertical polarization.

To experimentally verify the performance of the optimal random coding metasurface, a sample was fabricated and measurements were conducted, as described in the Method section. In addition, a metal sheet of equivalent geometry was investigated as a reference. Figure 10 provides the experimental data for vertically polarized incidence of varying angles. Note the test environment is not a completely anechoic chamber and the metal is lossy, energy of the high-frequency reflected electromagnetic waves decrease sharply. In addition, the dielectric constant of F4B become increasingly unstable with the increase of frequency, leading to the shifts of the resonance frequency. However, both the metal and metasurface RCS results have the same drop rate. Thus, the metasurface can be considered reasonably to hold the characteristic of RCS reduction. The figure indicates that RCS reduction of over 10 dB is generated in the frequency range from 17 GHz to 40 GHz, and comparison between Figs 9(b) and 10(b) demonstrate generally good agreement between the simulation and experimental results in terms of the overall trends. While the metasurface unit cell exhibits phase aberrations at various angles of incidence, the RCS reduction decreases only slightly. However, overall, the proposed metasurface performs well.

## Discussion

In this paper, a broadband and broad-angle polarization-independent random coding metasurface for RCS reduction has been proposed and verified through simulations and experiments. Based on diffuse reflection theory and the scattering pattern reshaping method, a GA was introduced to obtain the optimal coding matrix composed of arbitrarily placed “0” and “1” elements. Excellent agreement between the simulated and the measured results demonstrate that the optimal random coding metasurface can efficiently realize broadband RCS reduction more than 10 dB from 17 GHz to 42 GHz when the angle of incident waves varies from 10° to 50°. The characteristic of broad bandwidth and broad-angle of incident waves for RCS reduction makes it promising for electromagnetic cloaking in microwave regime.

## Methods

**Figure 11:**
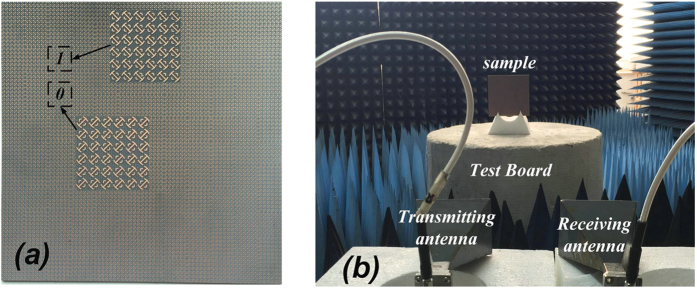
(a) The fabricated 1-bit GA-optimized coding metasurface. **(b)** The experimental setup for verifying RCS reduction.

The metasurface sample (shown in Fig. 11(a)) was placed at an equivalent height as the antennas in experiment, and the distance between sample and antennas was sufficient to avoid near field effects. The measurement setup is shown in Fig. 11(b). Pyramidal absorbing materials were placed around the sample to decrease background noise. In addition, a metal sheet of equivalent geometry was measured as a reference standard. To obtain the extent of RCS reduction, both the scattering coefficients of the metal and of the sample were measured. Two sets of ridged horn antennas were employed as transmitter and receiver, where both included frequency ranges of 15 GHz–18 GHz, 18 GHz–26.5 GHz, and 26.5 GHz–40 GHz. The antennas were connected to a vector network analyzer, which has the function of time domain gating. The receiver antenna can be reconfigured between transverse-electric and transverse-magnetic modes when rotated by 90°. The test bench allowed a 360° rotation to enable measurement of the RCS at various scattering angles.

## Additional Information

**How to cite this article**: Sun, H. *et al*. Broadband and Broad-angle Polarization-independent Metasurface for Radar Cross Section Reduction. *Sci. Rep.* **7**, 40782; doi: 10.1038/srep40782 (2017).

**Publisher's note:** Springer Nature remains neutral with regard to jurisdictional claims in published maps and institutional affiliations.

## References

[b1] Martín F. . Miniaturized coplanar waveguide stop band filters based on multiple tuned split ring resonators. IEEE Microw. Wireless Compon. Lett. 13, 511–513 (2003).

[b2] Garcia-Garcia Joan . Microwave filters with improved stopband based on sub-wavelength resonators. *IEEE Trans*. Microwave theory and techniques 53, 1997–2006 (2005).

[b3] Marqués R., Martín F. & Sorolla M. Metamaterials with Negative Parameters (Wiley, 2008).

[b4] Pendry John Brian. Negative refraction makes a perfect lens. Physical review letters. 85, 3966 (2000). 11041972 10.1103/PhysRevLett.85.3966

[b5] Pendry J. B. A chiral route to negative refraction. Science. 306, 1353–1355 (2004). 15550665 10.1126/science.1104467

[b6] Lezec Henri J., Jennifer A. Dionne & Harry A. Atwater. Negative refraction at visible frequencies. Science 316, 430–432 (2007). 17379773 10.1126/science.1139266

[b7] Barnes William L. Surface plasmon–polariton length scales: a route to sub-wavelength optics. Journal of optics A: pure and applied optics 8, S87 (2006).

[b8] Scalora Michael . Negative refraction and sub-wavelength focusing in the visible range using transparent metallo-dielectric stacks. Optics Express 15, 508–523 (2007). 19532269 10.1364/oe.15.000508

[b9] Kitamura Kyoko, Kyosuke Sakai & Susumu Noda. Sub-wavelength focal spot with long depth of focus generated by radially polarized, narrow-width annular beam. Optics express 18, 4518–4525 (2010). 20389464 10.1364/OE.18.004518

[b10] Milton Graeme W., Marc Briane & John R. Willis. On cloaking for elasticity and physical equations with a transformation invariant form. New Journal of Physics 8, 248 (2006).

[b11] Jiang Wei Xiang . Arbitrarily elliptical–cylindrical invisible cloaking. Journal of Physics D: Applied Physics 41, 085504 (2008).

[b12] Sun Jingbo, Ji Zhou & Lei Kang Homogenous isotropic invisible cloak based on geometrical optics. Optics express 16, 17768–17773 (2008). 18958058 10.1364/oe.16.017768

[b13] Wu Qun . Material parameters characterization for arbitrary N-sided regular polygonal invisible cloak. Journal of Physics D: Applied Physics 42, 035408 (2008).

[b14] Kildishev Alexander V. . Transformation optics: approaching broadband electromagnetic cloaking. New Journal of Physics 10, 115029 (2008).

[b15] Hu Jin, Xiaoming Zhou & Gengkai Hu. Design method for electromagnetic cloak with arbitrary shapes based on Laplace’s equation. Optics express 17, 1308–1320 (2009). 19188959 10.1364/oe.17.001308

[b16] Sun Jingbo . An extremely broad band metamaterial absorber based on destructive interference. Optics Express 19, 21155–21162 (2011). 22108966 10.1364/OE.19.021155

[b17] Ourir Abdelwaheb . Directive metamaterial-based subwavelength resonant cavity antennas–Applications for beam steering. Comptes Rendus Physique 10, 414–422 (2009).

[b18] Al-Joumayly Mudar A. & Nader Behdad. Wideband planar microwave lenses using sub-wavelength spatial phase shifters. IEEE Trans. Antenn. Propag. 59, 4542–4552 (2011).

[b19] Pendry John B., David Schurig & David R. Smith. Controlling electromagnetic fields. Science 312, 1780–1782 (2006). 16728597 10.1126/science.1125907

[b20] Schurig David . Metamaterial electromagnetic cloak at microwave frequencies. Science 314, 977–980 (2006). 17053110 10.1126/science.1133628

[b21] Jiang Wei Xiang . An ultrathin but nearly perfect direct current electric cloak. Applied Physics Letters 102, 014102 (2013).

[b22] Paquay Maurice . Thin AMC structure for radar cross-section reduction. IEEE Trans. Antenn. Propag. 55, 3630–3638 (2007).

[b23] Alu Andrea & Nader Engheta. Plasmonic and metamaterial cloaking: physical mechanisms and potentials. Journal of Optics A: Pure and Applied Optics 10, 093002 (2008).

[b24] Baumeier Björn, Tamara A. Leskova & Alexei A. Maradudin. Cloaking from surface plasmon polaritons by a circular array of point scatterers. Physical review letters 103, 246803 (2009). 20366219 10.1103/PhysRevLett.103.246803

[b25] Chen Pai-Yen & Andrea Alù. Atomically thin surface cloak using graphene monolayers. ACS nano 5, 5855–5863 (2011). 21662981 10.1021/nn201622e

[b26] Cui Tie Jun . Coding metamaterials, digital metamaterials and programmable metamaterials. Light: Science & Applications 3, e218 (2014).

[b27] Wang Ke . Broadband and broad-angle low-scattering metasurface based on hybrid optimization algorithm. Scientific reports 4, 5935 (2014). 25089367 10.1038/srep05935 PMC4120860

[b28] Gao Li-Hua . Broadband diffusion of terahertz waves by multi-bit coding metasurfaces. Light: Science & Applications 4, e324 (2015).

[b29] Holland J. H. Adaptation in Natural and Artificial Systems, University of Michigan Press, Ann Arbor, MI (1975).

[b30] Haupt Randy L. Thinned arrays using genetic algorithms. IEEE Trans. Antenn. Propag. 42, 993–999 (1994).

[b31] Haupt Randy L. An introduction to genetic algorithms for electromagnetics. IEEE Antenn. Propag. 37, 7–15 (1995).

[b32] Johnson J. Michael & Yahya Rahmat-Samii. Genetic algorithm optimization and its application to antenna design. Antennas and Propagation Society International Symposium, USA. IEEE. (1994, 20-24 June).

[b33] Marcano Diógenes. Synthesis of linear and planar antenna arrays using genetic algorithms. Antennas and Propagation Society International Symposium. IEEE. (1997, 13–18 July).

[b34] Siakavara Katherine. Novel fractal antenna arrays for satellite networks: Circular ring Sierpinski carpet arrays optimized by genetic algorithms. Progress in Electromagnetics Research. 103, 115–138 (2010).

[b35] Jain Rajashree & Mani G. S. Dynamic thinning of antenna array using genetic algorithm. Progress in Electromagnetics Research B. 32, 1–20 (2011).

[b36] Goswami Bipul & Durbadal Mandal. A genetic algorithm for the level control of nulls and side lobes in linear antenna arrays. Journal of King Saud University-Computer and Information Sciences 25, 117–126 (2013).

[b37] Khalid Amina . Synthesis of linear antenna array using genetic algorithm to reduce peak sidelobe level. ELECO 2015, Bursa, Turkey. Chamber of Electrical Engineers of Turkey. (2015, 26–28 Nov.).

[b38] Mosallaei Hossein & Yahya Rahmat-Samii. RCS reduction of canonical targets using genetic algorithm synthesized RAM. IEEE Trans. Antenn. Propag. 48, 1594–1606 (2000).

[b39] Zhu Xinyue . Design and optimization of low RCS patch antennas based on a genetic algorithm. Progress in Electromagnetics Research. 122, 327–339 (2012).

[b40] Yao Jing-Jing . A RCS Reduction Design of Object with Anisotropic Impedance Surface Using Genetic Algorithm. PIERS. 3, 588–592 (2010).

[b41] Su Pei . An Ultra-wideband and Polarization-independent Metasurface for RCS Reduction. Scientific reports 6, 20387 (2016). 26864084 10.1038/srep20387 PMC4750059

